# Quality of Life 6 Months after COVID-19 Hospitalisation: A Single-Centre Polish Registry

**DOI:** 10.3390/jcm12165327

**Published:** 2023-08-16

**Authors:** Maciej Koźlik, Maciej Kaźmierski, Wojciech Kaźmierski, Paulina Lis, Anna Lis, Weronika Łowicka, Marta Chamera, Barbara Romanowska, Jakub Kufel, Maciej Cebula, Marek Jędrzejek

**Affiliations:** 1 Division of Cardiology and Structural Heart Disease, Medical University of Silesia, 40-635 Katowice, Poland; kazmierski.maciej@gmail.com (M.K.); jedrzejekmarek@gmail.com (M.J.); 2 Faculty of Medicine and Health Sciences, Andrzej Frycz Modrzewski Krakow University, 30-705 Krakow, Poland; wkazmierski97@gmail.com; 3 Cardiology Students’ Scientific Association, Department of Cardiology, SHS, Medical University of Silesia, 40-635 Katowice, Poland; lispaulinab@gmail.com (P.L.); lis.anna9898@gmail.com (A.L.); weronikalowicka22@gmail.com (W.Ł.); marta.chamera@op.pl (M.C.); barbara.romanowska@gmail.com (B.R.); 4 Department of Biophysics, Faculty of Medical Sciences in Zabrze, Medical University of Silesia, 41-808 Zabrze, Poland; jakubkufel92@gmail.com; 5 Individual Medical Practice Maciej Cebula, 40-754 Katowice, Poland; maciejmichalcebula@gmail.com

**Keywords:** SARS-CoV-2 infection, COVID-19, quality of life, SF-36

## Abstract

Background: The COVID-19 pandemic, which affected the entire global population, had an impact on our health and quality of life. Many people had complications, were hospitalised or even died due to SARS-CoV-2 infection. The health systems of many countries had to radically change their way of functioning and scientists around the world worked intensively to develop a vaccine for the SARS-CoV-2 virus. Aim: The aim of this work is to assess the quality of life of patients who were hospitalised for COVID-19, using the SF-36 questionnaire. Methods: Between May and August 2022, we conducted a telephone assessment of quality of life in patients who were hospitalised for COVID-19 at the Temporary Hospital in Pyrzowice (Silesia, Poland), between November 2021 and January 2022. Results: Quality of life was significantly lower in women (*p* = 0.040), those with DM2 (*p* = 0.013), CKD (*p* = 0.041) and the vaccinated (*p* = 0.015). Conclusions: People with chronic kidney disease, diabetes mellitus and women had a lower quality of life after COVID-19 disease. However, people who were vaccinated for SARS-CoV-2 had a lower quality of life than non-vaccinated people did. This is possibly due to the higher mean age, and probably the higher disease burden, in the vaccinated group.

## 1. Introduction

At the turn of 2019/2020, the COVID-19 pandemic, caused by severe acute respiratory syndrome coronavirus 2 (SARS-CoV-2) became a global concern. As of 16 March 2023, there have been 6,462,369 confirmed cases of the disease in Poland and 760,360,956 globally [1]. The predominant symptoms include shortness of breath, fever, fatigue, cough, diarrhoea, loss of smell or headache, but in some cases the disease can lead to more serious complications such as acute respiratory distress syndrome (ARDS), myocardial damage or thrombotic symptoms [2,3,4]. Regardless of the severity of the course of infection, distant consequences have also been observed with varying frequency, including long-COVID-19 syndrome [5,6]. Long COVID includes symptoms such as shortness of breath, fatigue and cognitive impairment occurring continuously since the initial infection or appearing after three months and lasting a minimum of two months [7]. All these factors significantly affect the quality of life of patients who have experienced SARS-CoV-2 infection.

The intensive work of scientists from all over the world has led to the invention and introduction of vaccines with different mechanisms of action. Of these, the highest efficacy of 94.29% is demonstrated by vaccines based on RNA [8]. In Poland, 57,935,715 distributed doses of vaccine and 22,643,631 fully vaccinated persons were registered by 21 March 2023. One dose was received by 22,871,721 persons, two doses were received by 19,757,415 persons, three doses were by 192,285 persons and a booster dose was received by 15,114,294 persons [9].

In Poland, since the beginning of the pandemic, more than a dozen so-called ‘temporary hospitals’ (COVID-19 hospitals) have been set up to receive only patients with confirmed SARS-CoV-2 infection [10]. The majority of patients were in a severe general condition, with increased dyspnoea and reduced saturation.

With the length of the pandemic and the emergence of new data, increasing attention has been paid to the impact of surviving SARS-CoV-2 infection on patients’ quality of life. Various forms can be used for this purpose, including The Medical Outcomes Study 36-item Short-Form Health Survey (SF-36) in the Polish-language version. Interest in short-form health questionnaires arose when the relatively frequent refusal to complete long forms during the Health Insurance Experiment was noted, resulting in a loss of patients from follow-up [11]. Through this, a method has been developed that should take a few minutes of a telephone call. The form comprehensively assesses the patient’s perceived health status across broad domains of physical and emotional health. It focuses on evaluating eight quality of life indicators: physical functioning, role limitations due to physical health, pain complaints, a general sense of health, vitality, social functioning, role limitations due to emotional problems and a sense of mental health [12]. SF-36 has been used in a number of studies published in prestigious scientific journals on a variety of conditions, including rheumatoid arthritis, brain tumours, endometriosis and type 2 diabetes [13,14,15,16]. During the COVID-19 pandemic, it has also become useful for assessing quality of life after surviving this infection, examining, for example, the benefits of pulmonary rehabilitation [17]. The majority of previous studies focused on the assessment of quality of life among COVID-19 patients using different questionnaires such as EQ-5D-5L or WHOQOL-BREF.

The aim of our single-centre study is to evaluate the quality of life of patients who underwent SARS-CoV-2 infection and were hospitalised at the Pyrzowice Temporary Hospital for patients with COVID-19 at the multi-profile unit. We focused on a later period of time, 6 months after hospitalisation, to emphasise the long-term consequences on patients’ overall well-being after this infection, specifically in the Polish population of internal medicine patients.

## 2. Materials and Methods

### 2.1. Study Population

**Figure 1:**
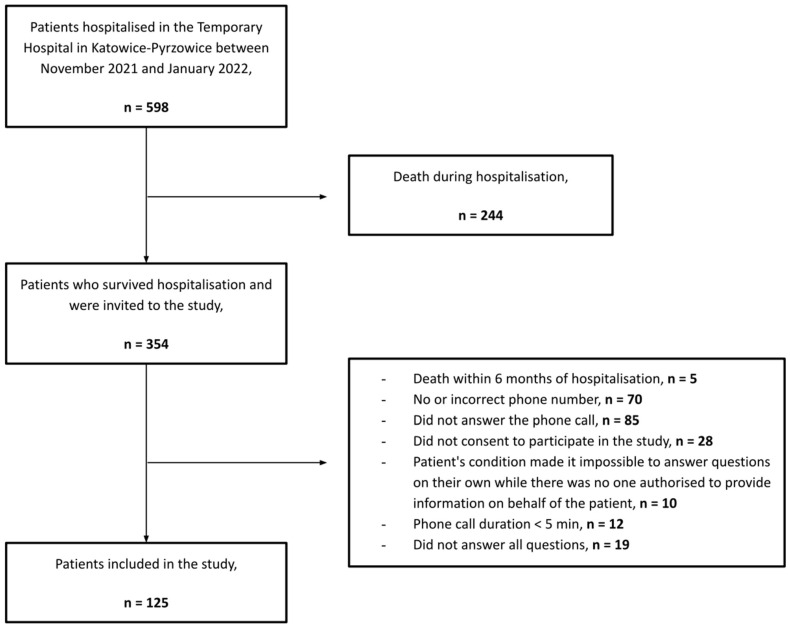
Study flowchart.

Between November 2021 and January 2022, 598 patients were hospitalised at the Temporary Hospital in Katowice-Pyrzowice, Poland, due to SARS-CoV-2 infection and required urgent hospitalisation in a specialised internal medicine unit for acute respiratory failure. In total, 354 patients (159 women and 195 men) who survived the hospitalisation were invited to participate in the study. Ultimately, 125 patients (54 women and 71 men) who met all inclusion criteria and did not meet the exclusion criteria were included in the study (Figure 1).

Telephone interviews were conducted between May and August 2022, during which questions from the SF-36 questionnaire were asked to subjectively assess quality of life 6 months after hospitalisation for SARS-CoV-2 infection. At the beginning of the telephone call, each patient was asked for consent to the survey. All procedures performed in the studies were in accordance with the 1964 Helsinki declaration and its later amendments or comparable ethical standards. Respondents were also asked about receiving a SARS-CoV-2 vaccine before and after hospitalisation and the number of doses taken.

The protocol for this study was approved by the Bioethics Committee of the Medical University of Silesia in Katowice (consent nr. PCN/CBN/0052/KB/103/22). Inclusion and exclusion criteria are presented in Table 1.

### 2.2. SF-36 Questionnaire and Score Calculations

The Polish version of the SF-36 (Short Form Health Survey) questionnaire [12], which was based on the English version [18], was used to assess quality of life (QOL). In the Polish version, the wording of 4 from 36 items was changed in 4 different questions. The SF-36 questionnaire is a subjective tool that allows a simple and quick assessment of QOL. It was designed so that the questions are understandable to the respondent and measure different aspects of quality of life. It allows for the study of both the general population and different groups of patients and is designed to meet the psychometric standards necessary for group comparison [19]. The SF-36 questionnaire and its Polish version are well-researched methods with proven reliability and validity [20,21,22].

The questionnaire consists of 11 questions with 36 statements that assess 8 quality of life indicators: physical functioning (PF; 10 items), role—physical (RP; 4 items), bodily pain (BP; 2 items), general health (GH; 5 items), vitality (VT; 4 items), social functioning (SF; 2 items), role—emotional (RE; 3 items), and mental health (MH; 5 items). The questionnaire contains two subscales, one assessing physical health based on PF, RP, BP and GH, and a subscale assessing mental health based on VT, SF, RE and PW. The respondent evaluates the statements based on the last 4 weeks. In addition, question 2 assesses current health status compared to that one year ago.

In the Polish version of the questionnaire, points are given when a dysfunction or limitation is present when assessing a statement. Questions 4 and 5 are answered in a dichotomous yes/no manner, and the remaining questions (1–3, 6–11) are rated on a 3-, 5-, or 6-point Likert scale. The Likert scale is a psychometric scale commonly used in questionnaire studies and allows for the differentiation of respondents’ attitudes. The respondent selects the answer on the scale that most closely matches how they feel. It is usually an unpaired scale in which the extreme values correspond to the most positive and negative responses and the middle response is neutral [23].

The total quality of life index is the sum of the scores for all 11 questions and ranges from 0 to 171 in the Polish version of the questionnaire. This provides an overall assessment of health status, where the highest scores indicate the lowest subjective level of quality of life. Questionnaires, complete scoring instructions and differences between the Polish SF-36 questionnaire and English SF-36 questionnaire are specified in the supplementary (Supplementary File S1).

### 2.3. Statistical Analysis

Quantitative and qualitative data were collected using Excel (version 16.75.2, Microsoft, Redmond, WA, USA), and statistical analysis was performed in Statistica 13.3 (StatSoft, Kraków, Poland). An initial, automatic analysis was performed to search for missing data and anomalous values. The assessment of the normality of the distribution of quantitative variables was performed with the Shapiro–Wilk test. In the absence of a normal distribution, the analysis of quantitative variables in relation to two-state grouping variables was performed using the Mann–Whitney U test. An attempt was made to evaluate the correlation of quantitative variables using the Spearman test and multi-parameter evaluation using the stepwise regression method without obtaining reliable results. The level of significance was *p* < 0.05.

## 3. Results

### 3.1. Characteristic of the Study Group

A total of 125 patients, including 71 men (56.8%), were qualified for the study on the basis of criteria defined by the SF-36 questionnaire. Among the patients who were in the study group, hospitalised at the Temporary Hospital in Katowice-Pyrzowice, many had a positive medical history. Sixty patients were confirmed to have hypertension (HA), which accounted for 48% of the study group. There was also a significant proportion of patients with ischaemic heart disease (IHD), numbered at 26, accounting for 20.8% of the total. A similar proportion comprised obese patients, with 27 patients (21.6%). There were 22 patients with chronic kidney disease (CKD), corresponding to 17.6% of patients in the study group. There were 20 patients with diabetes mellitus (DM), representing 16% of the study group. A positive oncological history was confirmed in 12 subjects (9.6%). Similarly, a history of stroke was frequently found in the study group, with 10 patients (8%). A smaller group of patients consisted of those with a positive pulmonary history, i.e., 11 patients with bronchial asthma, 4 patients with chronic obstructive pulmonary disease (COPD) and 3 patients with obstructive sleep apnoea (OBS), corresponding to 8.8%, 3.2% and 2.4% of the study group, respectively. The number of post-pulmonary embolism (PE) patients was 4 (3.2%). Nicotinism was reported by 12 patients (9.6%). The full list of characteristics of the study group is presented in the table below (Table 2).

Among all patients included in the study, 39 patients were vaccinated against COVID-19, representing 31.2% of the total, of whom 3 patients (2.4%) received a baseline dose and 2 booster doses, 33 patients (26.4%) received a baseline dose and 1 booster dose, and 3 patients (2.4%) received only the baseline dose of the vaccine. No patients received a third booster dose because there was still no opportunity to be vaccinated with a third booster dose during the period before and during hospitalisation and during the follow-up (Table 2).

The mean age of all patients vaccinated against COVID-19 was 67.15 ± 13.06 years. The mean age of patients vaccinated against COVID-19 before hospitalisation was 68.60 ± 11.35 years, and that of patients vaccinated against COVID-19 after hospitalisation was 67.31 ± 12.92 years. In comparison, the mean age of all patients not vaccinated against COVID-19 was 60.35 ± 14.62 years. The mean age of patients not vaccinated against COVID-19 before hospitalisation was 64.57 ± 15.81 years, and that of patients vaccinated against COVID-19 after hospitalisation was 66.85 ± 13.86 years.

### 3.2. Main Findings

Statistical analysis was performed. The female:male gender ratio in the study group was 1:1.31. The study showed that women had significantly higher SF-36 scores (*p* = 0.040) compared to men, i.e., women received an average of 69.50 ± 35.63 points and men received an average of 56.65 ± 32.02 points on the SF-36 questionnaire. The study also found that patients with a history of CKD had significantly higher SF-36 scores (*p* = 0.041) compared to those without a history of CKD. Patients with CKD had an average score of 74.18 ± 29.10, while patients without CKD had an average score of 50.96 ± 34.66. This research indicated that patients with DM had significantly higher SF-36 scores (*p* = 0.013) compared to patients without known DM. Those with a history of DM had a mean score of 77.15 ± 26.68 and those without a history of DM had a mean score of 59.35 ± 34.71. The study revealed that COVID-19-vaccinated patients had significantly higher SF-36 values (*p* = 0.015) compared to COVID-19-non-vaccinated patients. COVID-19-vaccinated patients had an average score of 73.05 ± 30.92, and COVID-19-non-vaccinated patients had an average score of 56.23 ± 34.34. Median values for statistically significant variables are presented in the form of diagrams in the supplementary (Supplementary File S2). While analysing the data, some disease entities and burdens turned out not to be statistically significant. All independent variables are presented below (Table 3).

## 4. Discussion

The study showed that COVID-19 vaccination does not improve quality of life after SARS-CoV-2 virus infection compared to that of non-vaccinated individuals. The study showed that people with CKD have significantly higher SF-36 scores, meaning that their QOL scores are worse than those without CKD. The study also found that people with DM have higher values on the questionnaire, which translates into a worse assessment of quality of life than that of people without DM. In the study, women’s quality of life scores are significantly lower than men’s.

Taboada et al. investigated quality of life in patients who underwent hospitalisation in an intensive care unit for SARS-CoV-2 infection. Patients assessed their quality of life and functional status 3–6 months before COVID-19 using the EuroQol Group Association’s five-domain, three-level questionnaire (EQ-5D-3L), which consists of two sections: a descriptive system and a visual analogue scale. They obtained results showing that 67% of the patients studied had a significantly lower quality of life after hospital discharge, mainly due to reduced mobility and pain or discomfort [24]. In our study, we examined patients who were hospitalised in the internal medicine ward and did not end up in the intensive care unit. The difference in the study group (patients hospitalised in the ICU vs. patients hospitalised in the internal medicine ward) may have influenced the difference in the patients’ quality of life scores as we suspect a more severe course of the disease in those who had to go to the ICU for this reason. In addition, these patients may have had an initially higher burden of concomitant diseases and may have been of older age than those hospitalised in an internal medicine ward. The EQ-5D-3L questionnaire was also used by Taboada et al. in their study, comparing quality of life and persistent symptoms after hospitalisation for COVID-19 among patients who required hospitalisation in an intensive care unit for COVID-19 and those who did not require intensive care hospitalisation. More ICU patients showed a worsening of their QOL compared to patients who were not hospitalised in an ICU ward (71.9% vs. 43.7%, *p* = 0.004). In total, 52.4% of all patients reported a worsening of at least one of the five dimensions analysed in the EQ-5D-3L, and 24% of all patients reported a worsening of two or more dimensions. More women than men reported problems with usual activities (25.0% vs. 12.1%, *p* = 0.024), pain or discomfort (45.2% vs. 26.3%, *p* = 0.007) and anxiety or depression (53.6% vs. 24.2%, *p* < 0.001) [25]. Similarly, in our study, women had significantly higher scores on the SF-36 and therefore had a lower quality of life than men did. As with the previous study, the differences in scores may have been due to the different study groups.

The EQ-5D-5L questionnaire was used by Tarazona et al. to assess quality of life in outpatients who had survived COVID-19. COVID-19 outpatients (*n* = 96) had significantly lower health-related quality of life than controls (*n* = 81) did one year after SARS-CoV-2 infection; the EQ-5D-5L index averaged 0.87 in examined cases and 0.95 in controls (*p* = 0.002) [26]. This work compared outpatients who developed COVID-19 to patients who were not diagnosed with COVID-19 disease. The difference in results between this work and those of Tarazona et al. can also be explained by the different selection of the study group—in our work, we only considered patients diagnosed with COVID-19 and investigated the impact of the disease on their quality of life after the disease. We did not compare this with a control group that had not contracted COVID-19 or had not been hospitalised for COVID-19 disease. Qu et al. also used the SF-36 questionnaire to assess the quality of life of patients after hospitalisation for COVID-19, in a version with scores ranging from 0 to 100 points. Apart from the ‘general health’ category, in all other categories included in the questionnaire, the study participants scored significantly lower (*p* < 0.001). Quality of life was assessed 3 months after discharge and compared to norms for the general Chinese population. In addition, men relative to women had a better quality of physical and mental health after hospital discharge [27]. Despite the similar timing of the QOL assessment in patients in this study, the QOL scores obtained were compared to the overall norms for the general population and not to quality of life before the disease, as in our study. This may have influenced the difference in results between the papers.

Navarro et al., who also used the EQ-5D-5L scale, observed a reduction in the quality of life of up to 56% in patients hospitalised with moderate to severe SARS-CoV-2 infection. In contrast to this work, Navarro et al. included patients as early as 30 days after the onset of first symptoms, whereas in our study, patients were not interviewed until approximately three months after hospitalisation at the earliest. Bearing in mind that COVID-19 disease is an acute viraemic infection, this may have influenced the final results of the study, as the severity of COVID-19 disease symptoms may be greater (and thus quality of life in such individuals may be visibly lower) in those newly recovered from the disease compared to those who have already undergone some convalescence [28]. Van der Sar-van der Brugge et al. used the SF-36 to assess HRQoL in 101 patients with SARS-CoV-2 pneumonia, classified as moderate or severe pneumonia according to WHO definitions. Six weeks after hospital discharge, significant deterioration was found in all SF-36 domains except pain (*p* = 0.0001). The domains with the greatest reductions were physical role limitation, physical functioning and vitality [29]. Similar to the work of Navarro et al. the timing of the QOL assessment occurred earlier than that in our study (6 weeks after discharge vs. 3 months after hospitalisation) which may have influenced the difference in results. Howlader et al. used the WHOQOL-BREF questionnaire to assess quality of life in 3244 randomly selected patients in Bangladesh. The study found significantly lower QoL in women, those with chronic concomitant diseases, the unemployed and the elderly. However, symptoms decreased each day after diagnosis [30]. Similarly, in our study, women and people with chronic diseases (such as DM2 or CKD) had a lower quality of life. It can also be hypothesised that the later the QOL assessment is carried out, the less it will be influenced by COVID-19-related symptoms and more by the patient’s current illnesses and situation, such as age, gender or chronic diseases. The EQ-5D-5L questionnaire was also used in Giao et al.’s study, with the addition of the EuroQoL-Visual Analogue Scale (EQ-VAS) score to determine self-assessed health status. Lower scores were reported among those aged 60 years and older, women, those with comorbidities, those with persistent symptoms, those who were living alone and those who were experiencing stress (all *p* < 0.001) [31]. Ayuso Garcia et al. also used the EQ-5D in combination with the EQ-VAS and EQ-Health. Both the EQ-VAS and EQ-Health Index were lower in women, patients older than 65 years, patients with comorbidities and those who required hospitalisation during acute SARS-CoV-2 infection [32]. In our study, similarly lower scores were obtained for people with diabetes, chronic kidney disease, women and vaccinated people who initially had more comorbidities and were older. In the study by d’Ettorre et al. using the EQ-5D-5L and EQ-VAS scales, female gender, unemployment status and chronic comorbidities were the most common predictors of having any problems in each EQ-5D-5L domain, and an older age and higher body mass index (BMI) were also found to be associated with lower EQ-VAS scores [33]. As in our study, lower quality of life was shown here among patients with pre-existing chronic diseases, including type 2 diabetes. 

It should be noted that the study analysed survivors of life-threatening conditions associated with severe respiratory failure due to SARS-CoV-2 virus infection. A large number of patients were ultimately not included in the study group due to death during hospitalisation itself and after the hospitalisation period. The status of having been vaccinated of this population was not assessed in this work.

Those vaccinated against COVID-19 who were included in the study described their health status by assessing their quality of life as worse than those who were not vaccinated.

However, the mean age of the unvaccinated patients was visibly lower than that of the vaccinated patients (60.35 ± 14.62 vs. 67.15 ± 13.06). Additionally, the number of concomitant diseases was higher in the vaccinated than in the unvaccinated group (2.44 ± 1733 vs. 1.13 ± 1170). We can therefore suspect that vaccinated persons present a lower quality of life than unvaccinated persons do due to a higher burden of concomitant diseases and an older age. In this study, the quality of life assessed by women was lower compared to that of men, which is confirmed by other studies. In the work by Lee, K.H. et al. and Badr, H.E. et al. that assessed quality of life, women also scored lower than men did [34,35]. In this work, patients with CKD and DM reported worse quality of life than did those who did not have the aforementioned disease entities.

In summary, other studies present similar findings of reduced quality of life after SARS-CoV-2 infection; however, they used either a different questionnaire or focused on another time point than those in our study. We concentrated on a specific period of time, 6 months after hospitalisation, to highlight the long-term implications on patients’ general condition after this disease. Moreover, most of the existing studies presented the results obtained from specific populations, such as the Chinese population in Qu et al.’s study or the Bangladeshi population in Howlander et al.’s study. We strongly believe that our results can be a valuable source of information about the QOL of COVID-19 survivors 6 months after hospitalisation in the Polish population.

## 5. Limitations

Several limitations of this study should be highlighted. Firstly, the study involved a small group of patients and all patients were treated at a single centre, so the results of this study should not be generalised. There were more unvaccinated patients in the study group than there were vaccinated patients, which may have influenced the QOL results obtained. Another limitation is that the QOL of patients was examined only once after 6 months of hospitalisation, so the data obtained do not describe the long-term status of patients and make it impossible to compare patients’ status over time. A very important problem is the lack of SF-36 norms for the Polish population, which makes it impossible to compare the results obtained to those of the general Polish population. In addition, conducting the survey by telephone resulted in the elimination of many patients from the study due to problems with correct contact details, availability or ability to respond. Finally, a disadvantage of surveying by telephone questionnaire is the inability to verify the veracity of patients’ answers and the lack of use of objective methods such as laboratory tests. A multicentre study including more follow-ups over several years would allow a more complete assessment of the QOL of patients after hospitalisation for COVID-19.

## 6. Conclusions

Our study showed that people with persistent diseases, such as chronic kidney disease or diabetes mellitus, had lower quality of life after SARS-CoV-2 infection. Moreover, women complained about a lower quality of life than that of men. However, people who were vaccinated for COVID-19 had a lower quality of life than did non-vaccinated people. This is possibly due to the higher mean age, and probably the higher disease burden, in the vaccinated group. More research is needed to further investigate this matter.

## Figures and Tables

**Table 1:** Inclusion and exclusion criteria.

Inclusion Criteria	Exclusion Criteria
Patient age ≥ 18 years old	Death of a patient within 6 months from the end of hospitalisation in the Temporary Hospital in Katowice-Pyrzowice
Call duration ≥ 5 min	Missing or incorrect contact telephone number of the patient
Verbal consent of the patient to a telephone interview	Failure to answer the phone by the patient (after 3 attempts, with an interval of 5 days between each attempt)
Obtaining answers to all questions in the SF-36 questionnaire during a telephone interview	Patient’s condition not allowing him/her to answer the SF-36 questionnaire on his/her own during the telephone interview (dementia; mental disorder) while there was no authorized person to provide information on behalf of the patient

**Table 2:** Characteristics of the study group.

Parameter	Value
Females	54 (43.2%)
Males	71 (56.8%)
Age	62.22 ± 14.61
Body Mass Index (kg/m^2^)	29.64 ± 12.62
Obesity	27 (21.6%)
Smoking	12 (9.6%)
Chronic obstructive pulmonary disease	4 (3.2%)
Asthma	11 (8.8%)
Pulmonary embolism	4 (3.2%)
Organic brain syndrome	3 (2.4%)
Chronic kidney disease	22 (17.6%)
Diabetes mellitus	20 (16.0%)
Hypertension	60 (48.0%)
Ischaemic heart disease	26 (20.8%)
Ischaemic stroke	10 (8.0%)
Cancer	12 (9.6%)
Vaccinated	39 (31.2%)
Vaccinated—1 dose	3 (2.4%)
Vaccinated—2 doses	33 (26.4%)
Vaccinated—3 doses	3 (2.4%)

**Table 3:** Analysed independent variables in the study group of patients.

Parameter	*p*-Value
**General**	
Sex	0.04
Obesity	0.838
Smoking	0.746
Vaccinated	0.015
**Diseases**	
Chronic obstructive pulmonary disease	0.581
Asthma	0.8
Pulmonary embolism	0.555
Organic brain syndrome	0.162
Chronic kidney disease	0.041
Diabetes mellitus	0.013
Hypertension	0.429
Ischemic heart disease	0.098
Ischemic stroke	0.458
Cancer	0.11

## Data Availability

The data presented in this study are available on request from the corresponding author. The data are not publicly available due to privacy reasons.

## Supplementary Materials

The following supporting information can be downloaded at https://www.mdpi.com/article/10.3390/jcm12165327/s1, File S1: Differences between Polish and English version of SF-36 questionnaire; File S2: Diagrams with median values for statistically significant variables.
