# Supplementary material for: Quality of Life 6 Months after COVID-19 Hospitalisation: A Single-Centre Polish Registry
Source: J Clin Med. 2023 Aug 16;12(16):5327. doi: 10.3390/jcm12165327 (PMC10455592; doi:10.3390/jcm12165327)
Supplement: Supplementary file 1 [file jcm-12-05327-s001.zip › jcm-2511962-supplementary.pdf]

Supplementary File 1 (S1) – Differences between the Polish and English version of SF-36 questionnaire

Below we present the following issues:

A. Differences in the content of English version of SF-36 questionnaire and Polish version of SF-36 questionnaire
B. Differences in the scoring of English version of SF-36 questionnaire and Polish version of SF-36 questionnaire

A. DIFFERENCES IN THE CONTENT BETWEEN ENGLISH VERSION OF SF-36 QUESTIONNAIRE AND POLISH VERSION OF SF-36 QUESTIONNAIRE

In Polish version of the SF-36 questionnaire wording of 4 items differs from English version, below we present those items translated and compared to the English version.

Item 14:

- In Polish version: During the past month, have you had any of the following problems with your work or other regular daily activities as a result of your physical health: worse well-being than you expected?
  YES/NO
- In English version: During the past 4 weeks, have you had any of the following problems with your work or other regular daily activities as a result of your physical health? Accomplished less than you would like:
  YES/NO

Item 21:

- In Polish version: How many times have you had bodily pain during the past month?
  Never/Very rarely/Rarely/Occasionally/Often/Very often
- In English version: How much bodily pain have you had during the past 4 weeks?
  None/Very mild/Mild/Moderate/Severe/Very severe

Item 33:

- In Polish version: How true or false is each of the following statements for you? My health state is better than other people I know.
  Definitely true/Mostly true/Don't know/Mostly false/Definitely false
- In English version: How TRUE or FALSE is each of the following statements for you? I seem to get sick a little easier than other people.
  Definitely true/Mostly true/Don't know/Mostly false/Definitely false

Item 34

- In Polish version: How true or false is each of the following statements for you? I am more healthy than other people i know.
  Definitely true/Mostly true/Don't know/Mostly false/Definitely false
- In English version: How TRUE or FALSE is each of the following statements for you? I am as healthy as anybody I know.
  Definitely true/Mostly true/Don't know/Mostly false/Definitely false

The rest of the questions is the same in the English and Polish version of the questionnaire.

B. DIFFERENCES IN THE SCORING OF ENGLISH VERSION OF SF-36 QUESTIONNAIRE AND POLISH VERSION OF SF-36 QUESTIONNAIRE

In Polish version of the questionnaire points are admitted when a dysfunction or limitation is present when assessing a statement, so the highest scores indicate the lowest quality of life, while in English version higher score defines a more favourable health state.

In Polish version of the SF-36 questionnaire, original responses to individual items are recoded on a 0-4 or 0-5 range, not a 0-100 range as in the English version.

In Polish version average scores for 8 subscales and 2 summary scores are not calculated, in English version they are calculated and range from 0 to 100.

The quality of life index calculated in Polish version is the sum of all scores obtained and ranges from 0-17, there is no single overall score for the English version.

Supplementary File 2 (S2) - Diagrams with median values for statistically significant variables

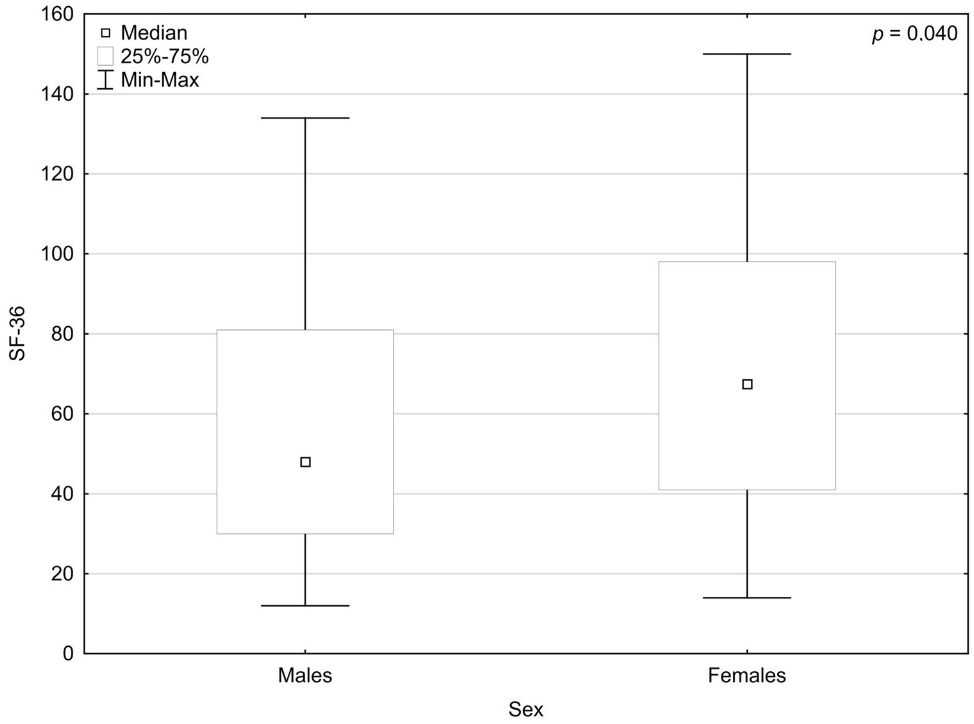

Figure S1. Comparison of SF-36 results by gender.

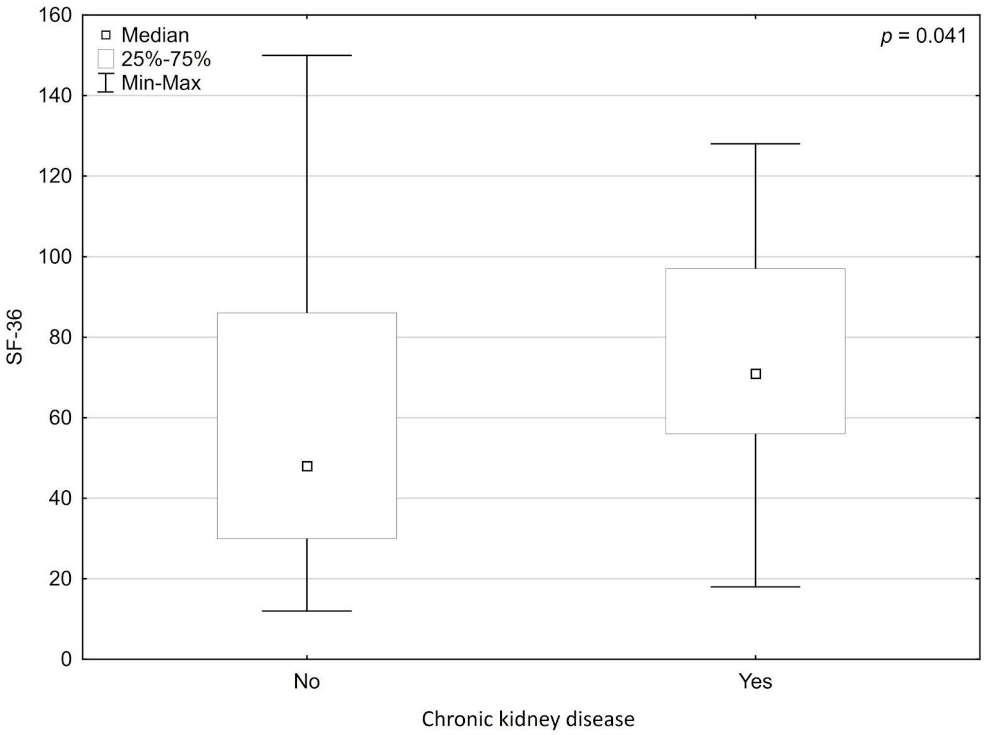

Figure S2. Comparison of SF-36 scores among patients with and without a history of CKD.

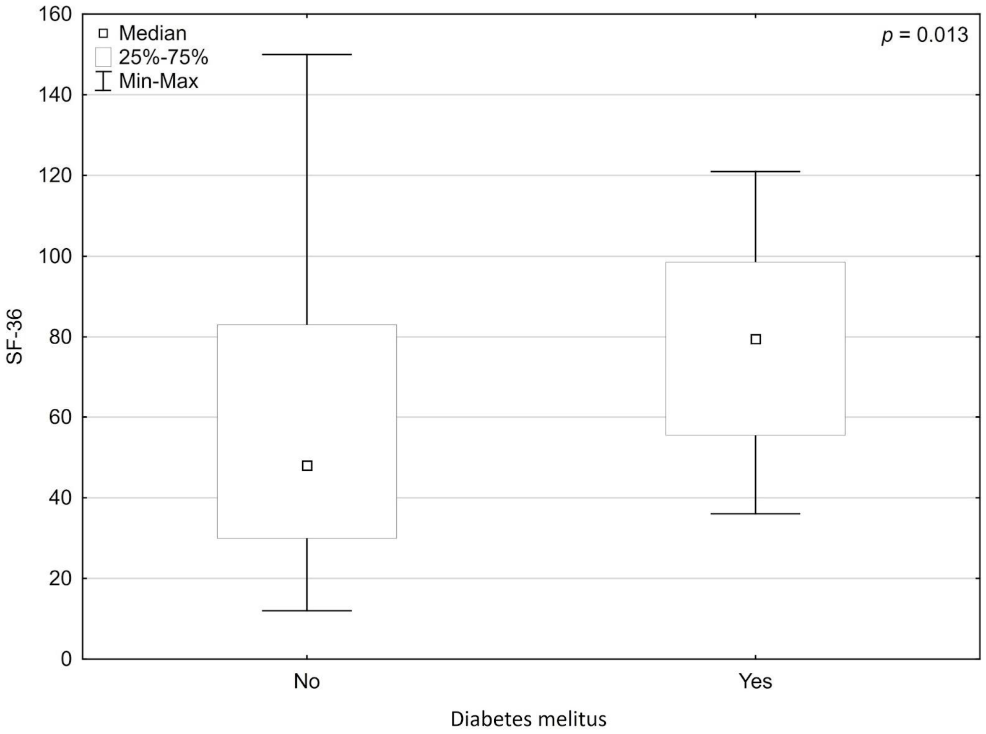

Figure S3. Comparison of SF-36 scores in patients with and without established diabetes mellitus.

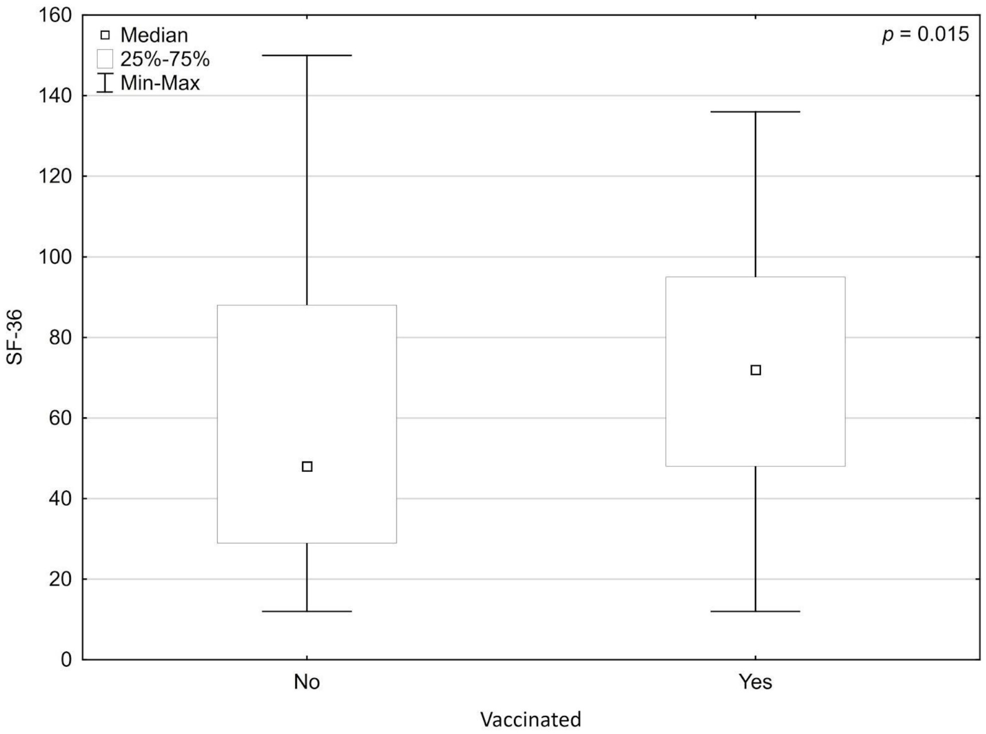

Figure S4. Comparison of SF-36 results in COVID-19 vaccinated and unvaccinated patients.
